# Supplementary material for: A Comparison of the Australian Dietary Guidelines to the NOVA Classification System in Classifying Foods to Predict Energy Intakes and Body Mass Index
Source: Nutrients. 2022 Sep 23;14(19):3942. doi: 10.3390/nu14193942 (PMC9571644; doi:10.3390/nu14193942)

Supplementary Table S1: Proportion of energy from all foods reported in the National Nutrition and Physical Activity Survey classified by the NOVA classification system and the Australian Dietary Guidelines

		NOVA classification				***ADG***	
Minor food groups	**Energy (%)**	**MP**	**CI**	**PF**	**UPF**	**FFG**	**DF**
Total	**100**	37.2	7.1	15.5	40.4	63.6	36.5
Regular breads, and bread rolls	**9.5**	0	0	64.6	35.4	100	0
Breakfast cereals	**3.7**	15.1	0	0	84.9	96	4
Mixed dishes where cereal is the major ingredient[1]	**2.3**	0	0	0	100	80	20
Margarine, table spreads and dairy blends	**1.1**	0	0	0	100	76	24
Fruit and vegetable juices, and drinks	**1.7**	6.2	0	0	93.8	55	45
Yoghurt	**1.1**	27	0	0	73	100	0
Flavoured milks and milkshakes	**0.6**	0	0	0	100	100	0
Pasta and pasta products (without sauce)	**2.8**	78.9	0	0	21.1	100	0
Savoury biscuits	**1.1**	0	0	0	100	53	47
Mixed dishes, predominantly poultry, beef, sheep, pork or mammalian game[2]	**1.1**	27.2	0	0	72.8	69	31
English-style muffins, flat breads, and savoury and sweet breads	**1.4**	0	0	42.3	57.7	78	22
Cheese	**3.1**	0	0	87.7	12.3	100	0
Meat and dairy substitutes	**0.4**	0	0	0	100	97	3
Cordials, electrolyte, energy, fortified drinks and beverage flavourings	**1.4**	0	0	0	100	18	82
Batter-based products[3]	**0.4**	0	0	0	100	47	53
Dietary and infant foods, intense sweeteners, cooking aids, extracts, essences	**0.4**	0	0.5	0	99.5	40	60

Other vegetables and legumes	**3.5**	86.4	0	8.8	4.8	100	0
Custards	**0.1**	0	0	0	100	100	0
Packaged soups, sauces, pickles, gravies, stocks, dips, salad dressings and herbs	**2**	1.9	1.1	4	92.9	14	86
Cakes, muffins, scones, cake-type desserts	**2**	0	0	0	100	4	96
Nuts, seeds and nuts and seed products	**1.9**	58.8	0	38.7	2.5	100	0
Potatoes	**2.8**	44.9	0	0	55.1	46	54
Flours and other cereal grains and starches	**4.8**	99.6	0	0	0.4	100	0
Beef, sheep, pork, mammalian meat and offal unprocessed	**4.9**	99.8	0	0	0.2	100	0
Tea, coffee, coffee substitutes	**0.4**	71.2	0	0	28.8	73	27
Dairy milk (cow, sheep and goat)	**6.3**	99.4	0	0.6	0	100	0
Fruit	**4.6**	96.7	0	3.3	0	100	0
Poultry and feathered game	**3.5**	100	0	0	0	100	0
Pastries	**2.3**	0	0	0	100	0	100
Soft drinks, and flavoured mineral waters	**2.1**	0	0	0	100	0	100
Chocolate and chocolate-based confectionery	**1.8**	0	0	0	100	0	100
Frozen milk products and other milk-based desserts	**1.8**	0	0	0	100	0	100
Sweet biscuits	**1.7**	0	0	0	100	0	100
Plant oils	**1.5**	0	100	0	0	100	0
Potato, corn, extruded, reformed and other savoury snacks	**1.5**	1	0	0	99	1	99
Eggs	**1.1**	100	0	0	0	100	0
Confectionary, sweet sauces and spreads and other sugar-based dishes	**0.7**	0	0	0	100	0	100
Muesli or cereal style, fruit, nut and seed-bars	**0.6**	0	0	0	100	0	100

Spirits and other alcoholic beverages	**0.6**	0	0	0	100	0	100
Fish and seafood	**1.8**	58.3	0	28.8	12.9	83	17
Cider and perry	**0.1**	0	0	100	0	0	100
Butter and Cream	**1.4**	0	98.8	0	1.2	0	100
Processed meats and sausages	**3.0**	0	0	48.1	51.9	3	97
Beers	**1.9**	0	0	100	0	0	100
Sugar, honey and syrups	**2.3**	12.4	79.6	0	8.1	0	100
Wines	**2.2**	0	0	100	0	0	100
Unspecified fats	**2.5**	0	91.3	0	8.7	0	100

1. *Pizza, sandwiches, kebabs, hot dogs, burgers, tacos, tortilla dishes, pasta and rice dishes, savoury dumplings and sushi*
2. *Casseroles, curries, and stir-fries*
3. *Pancakes, scones, waffles, donuts and crumpets*

Figure S1: Participant flow diagram from the National Nutrition and Physical Activity Survey

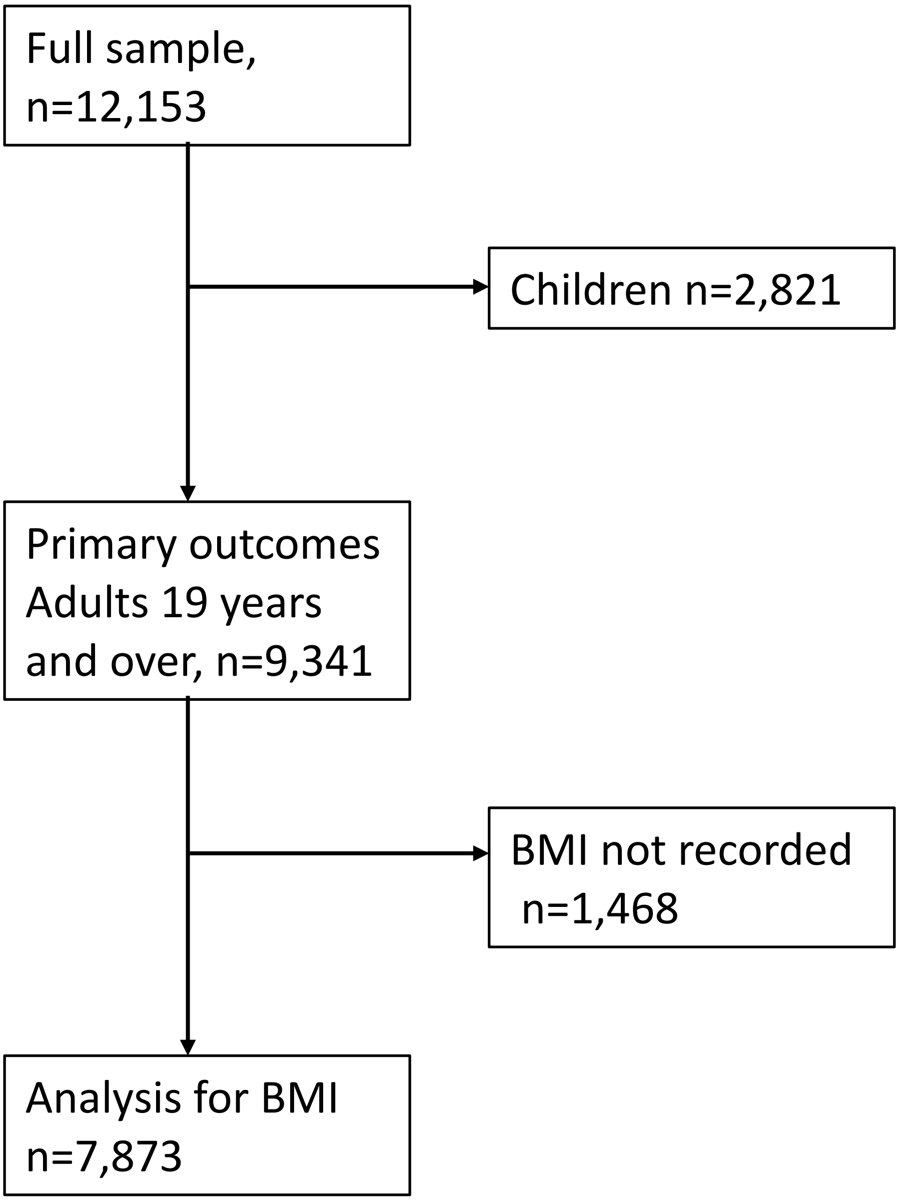

Supplement: Supplementary file 1 [file nutrients-14-03942-s001.zip › nutrients-1913113-supplementary.pdf]
